# An Account of Two Cases of Hydrocephalus Internus

**Published:** 1783

**Authors:** 


V. *An account of two cases of Hydrocephalus internus. By Mr.* Edward Wier, *Surgeon at Halifax in Nova Scotia. Communicated by Mr.* Thomas Young, *Surgeon to the Misericordia Hospital in London.*

THE remarkable fatality attending the twenty cases of Hydrocephalus internus, which were under the care of that justly celebrated brated phyſician, Dr. Whytt of Edinburgh, has, it is much to be feared, led many ſucceeding practitioners till of late too haſtily to adopt the idea of its incurable nature; in conſequence of which many under this diſeaſe have been abandoned to their fate, and many opportunities loſt of trying the effects of different remedies. The many facts lately produced in favour of a mercurial courſe determined me to try its effects in the two following caſes, which are the only ones I have met with in a very extenſive practice. As I noted every particular circumſtance both in the ſymptoms, and the effects of the remedies which appeared to merit attention, I ſhall lay them before the public juſt as they are ſtated in my diary; relying upon that candour, which is daily diſplayed by the humane part of the profeſſion, to every individual who diſcovers a deſire of adding his mite towards the improvement of a profeſſion, in which the happineſs of mankind is ſo eſſentially concerned.

Case I.—On the 5th of July 1781, I delivered M. Sharp at the full time of a female child. The labour was remarkably lingering, but at length terminated very favourably both to the mother and child.—At the time of the birth I thought the head appeared rather larger than than usual, but as no particular symptoms of disease presented, nothing respecting the child was either done or mentioned.—On the 7th of August following the mother brought the child to me for my advice. She informed me that its head had gradually increased from the birth to the present time; but that within the last four days its increase had been very rapid. I found the ossa bregmatis at the distance of three inches from each other in the course of the sagittal suture, and an evident fluctuation within the cavity. As the great weight of the head rendered it insupportable to the child, it was constantly supported upon a pillow. The child appeared most of the time in a heavy and sleepy state. At times it would suddenly start and scream out, as if in the most violent pain. The pupils of the eyes were remarkably dilated, and were much less affected by a strong light upon opening the lids suddenly than is common in health. Very frequent spasms in the limbs were observed, and the pulse was never below 160. The urine and stools were rather deficient, but the countenance still retained a natural and tolerably healthy appearance, and it took the breast well.

The nature of the cafe was obvious, but the degree led me early to guard myfelf by a prognoftic of a fatal termination. Being willing, however, to try a plan that had been attended with fuccefs in fomewhat fimilar difeafes, I began with *calomel.* gr. j. morning and evening, and directed ten grains of *ung. merc. fort.* to be rubbed in upon its legs twice a day. A great variety of fymptoms appeared from this time to the 27th of the fame month, when it died. Through the whole of this time it never took lefs than three or four grains of calomel, and had ℨß of the ointment rubbed in every day; but without any one evident effect upon either the bowels or mouth. Towards the laft ftage of the difeafe the child was frequently affected with convulfions, which were always followed by a comatofe and infenfible ftate for fome hours. But at length on the 27th of Auguft its fufferings were ended by death.

I was permitted to examine the head, and obferved the following circumftances:—After removing the whole of the integuments I found a defect of bone at leaft four inches fquare between the offa bregmatis and between thefe and the os frontis. This fpace was occupied by a firm, thick membrane, upon puncturing which

I drew off three full pints of a colourlefs and taftelefs fluid, perfectly refembling fpring water. The cerebrum was of its natural colour, but was compreffed into the fize of a fmall hen's egg. It was fupported by the tentorium, and was fo firm in its texture as to refift the knife almoft as much as a common kidney. Its different parts were eafily demonftrated, except the ventricles, which were wholly obliterated. The cerebellum was in a natural ftate, as were all the vifcera, but the body was emaciated in a very uncommon degree.

CASE II.—M. Cunningham was to all appearance a remarkably healthy, fine child at the time of his birth; and till he was about fifteen months old never difcovered the leaft fymptom of difeafe. He was then attacked with a train of complaints which were fuppofed to proceed from worms. The cafe was treated accordingly, and the child foon recovered. From that time to the prefent July 1782 (and he is now three years and three months old) he has had no complaint except now and then, when the wind has been to the eaftward, a flight degree of a pulmonary diforder, which has affected all the children of the fame family when young, and generally yielded to a change of air and fimple

ſimple remedies. About two months ago the child was obſerved by its mother to be reſtleſs in the night, with a ſlight degree of delirium, and conſiderable fever. Theſe complaints moderated, but did not leave him. About twelve days ago, his health appearing upon the decline, I was deſired to ſee him. I found him remarkably heavy and dull, with a low, remitting fever, which generally returned with the greateſt violence towards night. He would frequently raiſe his hand to his head, and was generally very drowſy, almoſt conſtantly picking his noſe. His countenance was pale and ſunk; he was much troubled with coſtiveneſs, and diſcharged but little urine. His tongue was covered with a thick white coat. His pulſe were very ſmall and quick (140) with a dry hot ſkin. Notwithſtanding all theſe ſymptoms of diſeaſe he never loſt his appetite; and though he vomited almoſt every thing he took, he would crave for food immediately after it. I begun at once to ſuſpect the diſeaſe to be a caſe of Hydrocephalus internus; and was determined to treat it accordingly.

As ſtools were wanting I began with preſcribing *pulv. rhei*, gr. viij. *calomel.* gr. iij. *mane ſumend.* He threw this medicine up; but upon repeating the ſame doſe with the addition of two drops of laudanum he retained it, and in the courſe of ſix hours got one ſmall, fœtid ſtool. As the ſymptoms remained, and he had got but one, and that a very offenſive diſcharge, I repeated the above purge the next day, yet he had but one ſmall ſtool from it. I now obſerved additional ſymptoms, which in my opinion put the nature of the caſe out of doubt. His eyes were turned inwards towards his noſe, ſo as to exhibit a degree of ſtrabiſmus ſeldom to be met with. The pupils of his eyes were remarkably dilated, his upper extremities were conſtantly agitated with involuntary catchings, and at one time his mouth was ſo firmly cloſed by ſpaſms, as to lead his mother to think he had a locked-jaw; but this ſymptom held him only a few minutes. He now appeared to have loſt the power of raiſing his hands to his head, and when placed upon his feet was not able to ſupport himſelf alone. His ſpeech faultered in ſuch a manner as to render his words unintelligible, and through the whole time he had the *riſus cynicus*. Theſe ſymptoms, joined to the difficulty of procuring ſtools, the remitting fever with very quick pulſe, irritation of the noſe, and circumſtances enumerated above, led me to conclude, clude, that the difeafe really was what has been mentioned, and determined me to adopt the only method which appears to have been found ferviceable. I accordingly on the 8th of July gave three grains of calomel in a little fugar in the morning, and the fame quantity in the evening, and applied a blifter behind each ear.—In the courfe of this day he had frequent hiccup.

On the morning of the 9th I found him lying in bed in a moft profufe fweat, with a flufhed, hot countenance. His pulfe was rather flower than yefterday. He had had two very offenfive ftools from the fix grains of calomel. He frequently cried out as if in violent pain. The other fymptoms were as before. As the blifter difcharged freely, it was left on, and three grains of calomel were ordered to be given morning, noon, and night.

July 10th. Has paffed a very reftlefs night. Pulfe very flow, low, and irregular. Has had only one ftool the laft 24 hours, and difcharged very little urine. The fquinting is a little abated, and he frequently moves his left hand to the right fide of his head. The arm and leg on the right fide appear to have loft the power of motion more than the left, and what is rather remarkable is, that the right fide of his face, half his nofe, lips, lips, chin, &c. are covered with large drops of ſweat, though the other ſide of the face is perfectly dry. In the after part of the day the left ſide appeared more affected than uſual, and at times he would ſtretch and twiſt his body, and be ſlightly convulſed. The bliſter being almoſt dry, ordered it to be dreſſed with *ung. baſil.* quickened with cantharides, and to give him four grains of calomel every four hours.

11th. Tho' he has loſt the power of ſpeech, he appears to underſtand all that is ſaid, and by ſigns intimates that his mouth is ſore, although there is no appearance of an increaſe of the ſalivary ſecretion.—Laſt night he was very reſtleſs, and troubled with frequent cramps, which have drawn in the abdominal muſcles greatly. His pulſe are very low, and quicker than yeſterday. He has voided no ſtool, though he has taken 16 grains of calomel in the laſt 24 hours. Has voided but little urine. Skin moiſt. Appears eaſieſt in this ſtate, which we endeavoured to encourage by warm cloths, particularly as he frequently appeared to ſhake as if from cold, and yet the thermometer has not been below 70 in the laſt week. Gave him an injection, which procured one very fœtid ſlimy ſtool. His cheeks appeared a little ſwelled. Every other ſymptom as as before. In the ſtool were ſeveral hard lumps of fœces, and ſome very ſmall worms. After this ſtool, he appeared livelier than at any time before. Ordered the calomel to be repeated as before.

12th. The ſquinting is entirely gone ſince yeſterday. His countenance has its natural appearance, and his pulſe approach much nearer the healthy ſtandard. His appetite is good, and he is in every reſpect much better. Repeated the injection, as he had had no ſtool, and deſired four grains of calomel to be given every 6 hours as before.

13th. Reſted well laſt night. Pulſe calm. Skin ſoft, and has a healthy feel. His countenance nearly as in a ſtate of health. In the courſe of the night, when aſleep, he was obſerved to have a frequent and involuntary motion of his right hand towards his head. His pupils near the proper ſize. Has had one ſtool, and diſcharged an uncommonly large quantity of urine. Cannot ſpeak. No appearance of ſore mouth, or ſpitting. Repeat the calomel as before.

14th. Very reſtleſs laſt night. This morning he ſpeaks very diſtinctly, for the firſt time. Appetite good. The clyſter procured a green ſlimy ſtool, of a very offenſive ſmell. Urine in a proa proper quantity. In difcharging his urine, he appears to be in pain (probably from the blifter). He can now move all his limbs at pleafure, and appears to be mending apace. Repeat the calomel.

15th. Has paffed a reftlefs night. Begins to walk, with affiftance. Every fymptom is moderated. Repeat the calomel as before.

16th. Refted well laft night. Had one ftool without the clyfter. Appetite good. Fever gone, and he appears ftronger. From this time the mercurial was omitted, and ten grains of *pulv. cort. Peruv.* were given every three hours in its ftead. By continuing this method for five days, with the ufe of the flefh-brufh, proper diet, gentle exercife, &c. he was recovered to a perfect ftate of health, and appeared better than he had been for fome time before the attack of this very dangerous difeafe.

